# MicroRNAs: new players in acute myeloid leukaemia

**DOI:** 10.1038/sj.bjc.6605232

**Published:** 2009-08-11

**Authors:** V Havelange, R Garzon, C M Croce

**Affiliations:** 1 Department of Molecular Virology, Immunology and Medical Genetics, Comprehensive Cancer Center, The Ohio State University, Columbus, OH, USA; 2 Hematological Section, The Human Genetic Center, Université catholique de Louvain, Brussels, Belgium; 3 Division of Hematology and Oncology, Department of Internal Medicine, Comprehensive Cancer Center, The Ohio State University, Columbus, OH, USA

**Keywords:** microRNAs, acute myeloid leukaemia, miRNA profiling, molecular alterations

## Abstract

MicroRNAs (miRNAs) are short non-coding RNAs that have key functions in a wide array of critical cell processes, including haematopoiesis by regulating the expression of multiple genes. Aberrant miRNA expression has been described in acute myeloid leukaemia suggesting a role in leukaemogenesis. In this review we summarise the current knowledge.

Acute myeloid leukaemia (AML) arises from myeloid progenitor cells that are arrested at early stages of differentiation. It is a cytogenetically heterogeneous disorder with acquired recurrent chromosomal alterations detected in about 55% of adult patients, such as translocations (i.e. t(15;17); t(8;21); t(9;11)), inversions (i.e. inv(16)), deletions (i.e. del(7q)), trisomies (i.e. +8) and monosomies (i.e. −5/−7) (reviewed by Mrózek *et al*, 2004). Importantly, AML patients could be stratified according to the detected cytogenetic abnormalities in high-, intermediate- and low-risk cytogenetic groups (Mrózek *et al*, 2004). In the remaining 45% of cases of cytogenetically normal AML (CN-AML), a number of novel molecular abnormalities have been described, such as the internal tandem duplication (ITD) in the juxtamembrane domain or mutation in the second tyrosine kinase domain (TKD) of FMS-like tyrosine kinase 3 (*FLT3*) gene, mutations in the nucleophosmin (*NPM1*) gene, CCAAT/enhancer binding protein-*α* (*CEBPA*) gene and in the Wilms’ tumour gene and partial tandem duplication (PTD) of the mixed-lineage leukaemia (*MLL*) gene (reviewed by Mrózek *et al*, 2007). In addition to mutations, overexpression of *ERG* (v-ets erythroblastosis virus E26 oncogene homologue) and *BAALC* (brain and acute leukaemia, cytoplasmic) genes has been found in CN-AML (Tanner *et al*, 2001; Baldus *et al*, 2004). Like cytogenetics, molecular abnormalities in CN-AML not only have improved the classification of this now heterogeneous group of AMLs, but also have prognostic implications. For example, *NPM1* mutations predict favourable outcome in young CN-AML in the absence of *FLT3*-ITD (reviewed by Mrózek *et al*, 2007). The presence of *CEBPA* mutations identifies a group of patients with a better prognosis within the young high-risk CN-AML group (patients with *FLT3*-ITD and/or wild-type (wt) *NPM1*) (Marcucci *et al*, 2008b). Otherwise, CN-AML patients with *FLT3*-ITD have a significantly inferior outcome compared to patients with *FLT3*-wt (reviewed by Mrózek *et al*, 2007). The level of *FLT3*-ITD mutant allele was also correlated with outcome, whereas the prognosis impact of *FLT3*-TKD is controversial. *MLL*-PTD has been associated with shorter complete remission duration or worse event-free survival (EFS) without effect on overall survival (reviewed by Mrózek *et al*, 2007). Many of these prognostic studies are difficult to interpret because of the co-existence of one or two mutations which have to be taken in consideration because of their probable interactions and influence on prognosis. Additional limitations arise from the low number of patients and treatment differences (reviewed by Gaidzik and Döhner, 2008).

Despite great progress, AML biology remained poorly understood. Over the past years, complementary DNA (cDNA) microarrays have been used to interrogate whole gene expression in AML samples. Indeed, several groups have reported distinctive signatures associated with particular cytogenetic and molecular groups of AML (Bullinger *et al*, 2004; Ross *et al*, 2004; Valk *et al*, 2004; Radmacher *et al*, 2006). Using unsupervised analyses, we identified novel subgroups of AMLs based on gene expression profiling (GEP) obtained using Affymetrix microarrays. Moreover GEP allowed to further classify previously defined cytogenetic subgroups. For example, GEP uncovered substantial heterogeneity in core binding factor (CBF) AMLs suggesting alternative mutations or deregulated pathways involved in transformation (Bullinger *et al*, 2007). In some cases, the GEP signatures were able to predict outcome (Bullinger *et al*, 2004; Ross *et al*, 2004; Valk *et al*, 2004). More importantly, a cDNA microarray study identified a gene expression signature that separated CN-AML into two prognostically relevant subgroups (Bullinger *et al*, 2004). The prognostic value of this signature was validated in a different set of CN-AML patients using a different microarray platform (Radmacher *et al*, 2006). These studies confirmed the possible applicability of GEP for outcome prediction in CN-AML. Despite this progress, focusing on known genes will likely not suffice to uncover the molecular puzzle of AML. The integration of a whole genome approach including non-coding RNAs may lead to an improved understanding of AML biology.

MicroRNAs (miRNAs) are non-coding RNAs of 19–25 nucleotides in length that regulate gene expression by repressing translation or accelerating mRNA decay (Bartel, 2004). MicroRNAs are involved in critical biological processes, including development, cell differentiation, apoptosis, proliferation and haematopoiesis (Chen *et al*, 2004; Poy *et al*, 2004; Xu *et al*, 2004; Cheng *et al*, 2005; Karp and Ambros, 2005). Recent data indicate that miRNAs are deregulated in diseases, such as diabetes, heart disease and cancer (Calin *et al*, 2002; Poy *et al*, 2004; van Rooij *et al*, 2006). The first study connecting miRNAs and leukaemia reported frequent down-regulation of the miRNA cluster; *miR-15a/miR-16-1* in chronic lymphocytic leukaemia (CLL) (Calin *et al*, 2002). This cluster is localised at chromosome 13q14.3, a genomic region, which is deleted in about 65% of CLL patients. Further work showed that *miR-15a/miR-16-1* cluster targets the anti-apoptotic *BCL-2* (Cimmino *et al*, 2005). Thereby, this study provides with an alternative explanation for the *BCL-2* up-regulation observed in CLL. Following earlier reports, two large miRNA profiling studies confirmed the widespread deregulation of miRNAs in cancer (Lu *et al*, 2005; Volinia *et al*, 2006). But how miRNAs contribute to oncogenesis? Further research established that miRNAs can behave as tumour suppressor genes or oncogenes (Calin and Croce, 2006; Garzon *et al*, 2006). An miRNA, which is down-regulated and targets an oncogene, acts as a tumour suppressor gene. For example, *let-7a*, which is down-regulated in lung adenocarcinoma, targets the highly expressed oncogene *K-RAS* (Johnson *et al*, 2005). In contrast, an miRNA which is over-expressed and targets a tumour suppressor gene acts as an oncogene (Calin and Croce, 2006; Garzon *et al*, 2006). For example, high expression of *miR-155* and its host gene, the non-coding RNA B-cell integration cluster (*BIC*) has been reported in different human B-cell neoplasms suggesting a role for *miR-155* in B-cell lymphomagenesis (Eis *et al*, 2005). This hypothesis was later confirmed by Costinean *et al* (2006) who reported an *miR-155* transgenic mouse developing a polyclonal pre-leukaemic pre-B-cell proliferation evident in the spleen and bone marrow (BM) followed by a full-blown malignancy.

Over the past 3 years, several groups have described miRNA signatures associated with recurrent cytogenetic abnormalities, molecular aberrations and outcome in AML. In this review we discuss the current miRNA profiling studies in AML and outline future prospects and potential therapeutic applications.

## miRNA expression in AML blasts, stem cells and committed progenitors

Four recent large-scale studies have reported miRNA expression in primary AML blasts and CD34+ selected cells (Garzon *et al*, 2008a; Jongen-Lavrencic *et al*, 2008) or BM samples (Dixon-McIver *et al*, 2008; Li *et al*, 2008) from healthy donors. Garzon *et al* (2008a) identified 26 down-regulated miRNAs and none up-regulated miRNAs in 122 newly diagnosed AML samples compared with BM CD34+ cells from 10 normal donors using a custom microarray platform. This is consistent with the miRNA profiling results obtained from 334 tumours, including leukaemias, and normal tissues using a bead-based flow cytometric miRNA platform (Lu *et al*, 2005). Lu *et al* (2005) found in general a lower expression of miRNAs in tumours compared with normal tissues. They hypothesised that global miRNA expression might reflect the state of cellular differentiation. Indeed, they observed in haematopoietic progenitor cells undergoing erythroid differentiation an increase in miRNA expression at a later stage of differentiation (Lu *et al*, 2005).

A second AML study analysed 215 newly diagnosed leukaemic patients and CD34+ cells from four healthy subjects using a multiplexing real-time quantitative polymerase chain reaction (qRT-PCR) method (Jongen-Lavrencic *et al*, 2008). Only 11 miRNAs (5 up- and 6 down-regulated) were found differentially expressed in normal CD34+ specimens with respect to the leukaemias. Among them, *miR-21* was found up-regulated in AML samples compared with CD34+ cells.

The two other studies compared miRNA expression in AML samples with BM from healthy donors. Dixon-McIver *et al* (2008) showed the significant deregulation of 33 miRNAs (17 up-regulated and 16 down-regulated) in 100 leukaemia samples with respect to two BM samples using a qRT-PCR assay. Li *et al* (2008) using bead-based miRNA expression profiling assays analysed 54 AML samples and 3 normal BM samples. Normal control samples were grouped together in a subcluster with unsupervised analysis.

Only few miRNAs were commonly (in at least two studies) down-regulated in AML patients, such as *miR-29b* and *miR-126*. Many factors might be involved in the disparities of the results from these studies. First, the purity of the leukaemic cells might influence the results. There could be differences in the blast percentage among the studies that could impact on the profiling. In one study, selection purity was improved by CD3/CD19 depletion after Ficoll separation of mononuclear cells. Second, the nature of the control sample is critical and can also explain the discrepancies. Two studies used BM CD34+ cells whereas the other two used whole BM or mononuclear cells. Third, other factors such as biology differences in the AMLs included in the studies, number of control samples, processing, platform and bioinformatics approaches (data normalisation, filtering and clustering) may influence the results. Further studies will be needed using highly pure populations, including CD34+ selected leukaemic cells, to assess miRNA changes with respect to CD34+ normal cells.

## miRNA signatures associated with cytogenetic and molecular alterations in AML

Garzon *et al* (2008a)analysed the miRNA expression in a cohort of 122 newly diagnosed primary AML samples with intermediate and poor prognosis using a custom miRNA platform. The authors identified miRNA expression profiles closely linked to 11q23 translocations, trisomy 8 and *FLT3*-ITD (Tables 1 and 2). Among the miRNAs down-regulated in balanced 11q23 translocation patients, many are tumour suppressor miRNAs that target critical oncogenes, i.e., *miR-34b* (*CDK4* and *CCNE2*), *miR-15a* (*BCL-2*), the *let-7* family (*RAS*), the *miR-29* family (*MCL-1* and *TCL-1*) and *miR-372* (*LATS2*). Interestingly, *miR-155* was found up-regulated in AML patients with high white count and *FLT3*-ITD mutations. This miRNA has been recently described to block ‘*in vitro*’ human myeloid colony formation, halt megakaryopoiesis (Romania *et al*, 2008) and induce a myeloproliferative disease in mice (O’Connell *et al*, 2008). A second study by Jongen-Lavrencic *et al* (2008) reported miRNA profiles using a qRT-PCR platform in 215 AML patients that include among others good risk karyotypes such as t(15;17), t(8;21) and inv(16). Using a combination of unsupervised and supervised analyses the authors identified distinctive miRNA expression profiles associated with known AML cytogenetic subtypes (Tables 1 and 2). Among them, the tumour suppressor miRNAs *let-7b* and *let-7c* (known to target the oncogenes *RAS*) were found down-regulated in CBF leukaemias (Johnson *et al*, 2005). None of these deregulated miRNAs was localised in the re-arranged chromosomal regions. Finally, the authors determined the minimal set of miRNAs and mRNAs (by performing GEP on the same AML cohort) that could predict a particular genetic subtype of AML. The mRNA-GEP class predictor was accurate in predicting AML with t(15;17), t(8;21) or inv(16) as well as CN-AML samples with *NPM1*, *CEBPA* mutations and *FLT3-ITD*. Another study using a qRT-PCR assay reported miRNA signatures that correlate with the most frequent cytogenetic alterations (t(15;17), t(8;21), inv(16), 11q23 translocations) in 100 AML samples. In particular, acute promyelocytic leukaemias bearing a t(15;17) had a distinctive signature including the up-regulation of seven miRNAs located on chromosome 14q32 which is not implicated in the translocation (Table 1; Dixon-McIver *et al*, 2008). More recently, the study of Li *et al* using another genome-wide bead-based miRNA expression platform analysed 52 AML samples with common translocations including t(8;21), inv(16), t(15;17) and *MLL* rearrangements (Table 1).

We reported in Table 1 the most significant miRNAs deregulated in association with the different cytogenetic subtypes in these four studies. In particular, we showed common miRNAs found up- or down-regulated by at least two studies in each subtypes (Table 1).

Li *et al* determined the minimal number of miRNAs that can accurately discriminate AML subtypes. Two (*miR-126* and miR-126*), three (*miR-224*, *miR-368* and *miR-382*) and seven miRNAs (from the polycistronic *miR17-92* cluster) are sufficient to predict CBF AML, t(15;17) and *MLL* AML respectively, resulting in a diagnosis accuracy better than 94% (Table 1). Some of these differentially expressed miRNAs were also identified in the class predictor reported by Jongen-Lavrencic including miR-126* in CBF leukaemias or *miR-382* in t(15;17) AML.

Garzon *et al* (2008b)focused on CN-AML with *NPM1* mutation which is the most common genetic alteration in CN-AML. They profiled 85 *de novo* AML patients (55 with *NPM1* mutation and 30 with *NPM1* wt) and found a strong miRNA signature that distinguishes CN-AML with *NPM1* mutation from *NPM1* wt (Table 2). The up-regulation of *miR-10a* and *miR-10b* in *NPM1*-mutated AML clearly differentiate the two different forms. These results were confirmed by Jongen-Lavrencic *et al* and in a recent study of 189 older CN-AML (Becker *et al*, 2009). Among the other miRNAs up-regulated, three families of tumour suppressor miRNAs were involved: *miR-15a/16-1*, several *let-7* and *miR-29* members whereas *miR-204* and *miR-128a* were down-regulated (Garzon *et al*, 2008b). Concerning *CEBPA* mutations, Marcucci *et al* (2008b) reported an miRNA signature associated with the presence of the *CEBPA* mutation in CN-AML patients. Interestingly, *miR-181a* and *miR-181b* were up-regulated in *CEBPA*-mutated cases. These results are consistent with a *CEBPA* mutation miRNA signature reported by Jongen-Lavrencic.

## Prognostic role of mirnas in AML

Few studies have correlated miRNA expression changes with prognosis in AML. Garzon *et al* (2008a) first reported that in a relatively older AML cohort of patients (median age 59) with intermediate- and poor-risk cytogenetics, patients with high expression of *miR-199a* and *miR-191* had a significant shorter overall survival and EFS. These results were validated in a second cohort of 60 AML patients with similar characteristics using a different technology; qRT-PCR. These two miRNAs (*miR-191* and *miR-199a*) predicted outcome independent from other variables, including age and cytogenetics. Marcucci *et al* (2008a) recently showed an miRNA signature that correlated with EFS in young CN-AML patients (<60 years) with high-risk molecular features (defined by the detection of *FLT3-ITD* and/or an *NPM1* wt). This subgroup of AML represents more or less a third of young AML patients. The prognostic signature included *miR-181a* and *miR-181b* which were inversely correlated with risk of event and miR*-124*, *miR-128-1*, *miR-194*, *miR-219-5p*, *miR-220a*, *miR-320* which were positively associated with the risk of event. This prognostic signature was independent from other factors. Furthermore, using mRNA gene-chip microarray analysis, the authors showed an inverse correlation between the expression of *miR-181* and predicted target genes. Many of these genes encode for proteins involved in pathways of innate immunity mediated by Toll-like receptors (*TLR2*, *TLR4*, *TLR8*) and interleukin-1*β* (NOD-like receptors *CARD8*, *12*, *15* and *CASP1*). Activation of Toll-like receptors induces production of inflammatory cytokines through nuclear factor *κ*B and interleukin-1*β* promotes the survival and the proliferation of AML blasts. No miRNA was found to be associated with outcome in low-risk group (*NPM1* mutation alone) (Marcucci *et al*, 2008a). Both prognostic signatures need to be confirmed in large prospective studies before clinical applications. The different prognostic miRNA signatures between the two studies can be explained by two main factors. First, the median age between the two studies was different; 60.3 years (range 18–86) *vs* 45 years (range 19–45). This is critical because *FLT3-ITD* and *NPM1* prognostic implications are age dependent. *FLT3*-ITD has been shown to have a negative prognostic impact only in young AML patients (reviewed by Mrózek *et al*, 2007), whereas it is not prognostic in the elderly (Stirewalt *et al*, 2001; Garzon *et al*, 2008a). *NPM1* mutation seems to be an independent prognostic factor in young and older AML patients but only in the absence of *FLT3*-ITD in the young patients cohort (reviewed by Gaidzik and Döhner, 2008; Becker *et al*, 2009). Second, the cytogenetic/molecular subgroups studied were different. Garzon *et al* (2008a) studied intermediate- and poor-risk cytogenetic groups, whereas Marcucci *et al* (2008a) focused on high-risk CN-AML.

## miRNAs and *HOX* genes

*HOX* genes are transcription factors which are important in the regulation of early stages of haematopoiesis, including self-renewal of haematopoietic stem cells (Sauvageau *et al*, 1995). Five miRNAs are embedded in the *HOX* genes cluster – *miR-196b* in *HOXA* (at chromosome 7p15), *miR-10a* and *196a-1* in *HOXB* (at chromosome 17q21), *miR-196a-2* in *HOXC* (at 12q13) and *miR-10b* in *HOXD* (at 2q31) clusters. A distinctive correlation between the *miR-10a*, *miR-10b* and *miR-196-1* and the *HOXA/HOXB* gene expression was first reported in 30 AML patients suggesting a common regulatory mechanism (Debernardi *et al*, 2007). Kuchenbauer *et al* (2008) recently showed an up-regulation of all the miRNAs located in the *HOX* cluster in a murine leukaemia model (engineered by over-expression of *NUP98*/*HOXD1*3 fusion gene with the oncogenic collaborator *MEIS1*).

### *Npm1*, mirnas and *hox* genes

Interestingly, *miR-10a*, *10b* and *miR-196b* have recently been linked to molecular or chromosomal alterations in subtypes of AML, in particular CN-AML with mutated *NPM1*. Garzon *et al* (2008b) identified a statistically significant up-regulation *miR-10a*, *miR-10b* in CN-AML with *NPM1* mutation. The over-expression was correlated with high expression level of *HOX* genes (mainly *HOXB* genes) (Garzon *et al*, 2008b). These results were validated by other groups (Debernardi *et al*, 2007; Jongen-Lavrencic *et al*, 2008; Becker *et al*, 2009). Whether these miRNAs are merely bystanders or involved in leukaemogenesis is still under investigation. Among the miRNAs down-regulated in *NPM1*-mutated AML, *miR-204* directly targets *HOXA10* and *MEIS1* (Garzon *et al*, 2008b). Over-expression of *HOXA10* in murine haematopoietic stem cells was reported to perturb myeloid differentiation and to lead to AML (Thorsteinsdottir *et al*, 1997). Similar outcome was shown by over-expressing *HOXA9* and *MEIS1* in mice (Kroon *et al*, 1998). The data suggest that miRNAs may be responsible in part of the *HOX* up-regulation observed in *NPM1*-mutated AML.

### *MLL-* and *HOX*-embedded miRNAs

Popovic *et al* (2009) reported that *miR-196b* is over-expressed specifically in AML with *MLL* rearrangement. The *MLL* gene (located in 11q23) is commonly involved in chromosomal translocations (with more than 60 different fusion partners reported) responsible of AML. Leukaemias related with *MLL* are in most of the cases characterised by an over-expression of a subset of *HOX* genes including *HOXA9* (Rozovskaia *et al*, 2001). *MLL* binds to specific clusters of CpG islands of *HOXA9*, adjacent to *miR-196b*, and maintains its expression by protecting these clusters from DNA methylation (Erfurth *et al*, 2008). Recently, Popovic *et al* (2009) showed that *MLL* regulates *miR-196b* expression similarly to the surrounding *HOX* genes. Moreover, MLL fusion proteins cause up-regulation of *miR-196b* in primary BM cells (Popovic *et al*, 2009). *MiR-196b* over-expression seems to increase proliferation, survival and partially block the differentiation of normal BM haematopoietic progenitor cells suggesting importance of *miR-196b* in MLL leukaemias. It is noteworthy that the MLL fusion proteins have also been shown to interact directly with Drosha, the nuclear RNase III enzyme crucial in miRNA biogenesis (Nakamura *et al*, 2007).

## miRNAs and epigenetics

Recent data indicate that miRNAs are targets of aberrant epigenetics in AML. The ETO/AML1 is the fusion protein resulting from the t(8;21) translocation detected in about 15% of AML. The *in vitro* expression of ETO/AML1 in haematopoietic stem cells leads to the expansion of the myeloid progenitors and a pre-leukaemic state. Fazi *et al* (2007) showed that the binding of *AML1* to the *pre-miR-223* promoter is responsible for *miR-223* transcription activation. The ETO/AML1 protein can bind to this site and activate histone deacetylates and DNA methyltransferases, thereby blocking the transcription of *miR-223* by inducing histone deacetylation and increasing DNA methylation.

Our group recently reported that over-expressing *miR-29b* in AML cell lines and primary AML blasts down-regulates the expression of DNA methyltransferases; *DNMT1*, *DNMT3A* and *3B. MiR-29b* targets directly *DNMT3A* and *3B* and indirectly *DNMT1* through its activator Sp1. Restoration of *miR-29b* leads to global DNA hypomethylation and re-expression of tumour suppressor genes such as the cyclin-dependent kinase inhibitor *p15*^*INK4b*^ and the oestrogen receptor *ESR1* genes (Garzon *et al*, 2009).

## Discussion and future directions

In this review, we described four recent large-scale global miRNA profiling studies in AML. The differences among the miRNA signatures reported by these studies can be explained mainly by the use of various cytogenetic and molecular AML subgroups and by the type of platform used to interrogate miRNA expressions. The frequencies of cytogenetic/molecular abnormalities were clearly different among the studies. For example, Garzon *et al* focused on intermediate and poor prognostic cytogenetic groups of AML, whereas Jongen-Lavrencic included good risk subgroups such as t(8;21), inv(16) and t(15;17). Because most of the strategies to identify distinctive miRNA signatures were based on two-way comparisons (i.e. inv(16) *vs* all other AMLs), the heterogeneity of the other AMLs will impact greatly on the analysis. This bias is less evident when the authors focus on well-characterised cytogenetic and molecular subgroups of AML. Common miRNAs have been found deregulated in CN-AML cases with *FLT3*-ITD, *NPM1* and *CEBPA* mutated and in patients with t(15;17), inv(16) and t(8;21). These results are significant because they will provide with a set of validated miRNAs whose functions will be further investigated. These studies may lead to a better molecular classification, understanding of AML biology and discovery of novel targets for treatment. In addition to improving diagnosis, miRNA signatures may enhance current prognostic markers and help to better stratify patients for treatment. But before any clinical applications, miRNA profiling studies require to be extensively validated and more standard, reliable and reproducible platforms will need to be developed. Future miRNA expression studies should use very well cytogenetically and molecularly characterised AML subtypes to minimise biology heterogeneity. Generation of small RNA libraries and next-generation deep sequencing will enable not only to quantify the whole miRNA transcripts but also to obtain sequences information and discover new miRNAs.

We also believe that miRNAs studies have potential relevant therapeutic implications. Synthetic miRNAs or anti-miRNAs alone or in association with chemotherapy could be promising in future AML therapies. Synthetic miRNAs can be used to restore lost tumour suppressor miRNAs expression. The other strategy (so far more developed) is to silence oncogenic miRNAs (with ‘antagomirs’ or anti-miRNA oligonucleotides or locked nucleic-acid-modified oligonucleotides) which are over-expressed in AML (Krützfeldt *et al*, 2005). Moreover, the impact of some drugs, such as DNA-demethylating agents, on miRNAs expression is also another therapeutic approach to modulate miRNA expression. If miRNA-based therapy offers new perspectives, several issues have still to be considered such as off-target effects, chemistry considerations and target delivery. A better comprehension of the regulation and function of the miRNAs will be necessary to develop future miRNA-based new drugs.

## Conclusion

Altogether, the data suggest that miRNAs may complement and enhance our current knowledge about AML. MicroRNA expression profiling has been associated with cytogenetic and molecular subtypes of AML. Functional studies suggested miRNAs may play a role in AML pathogenesis being involved in essential pathways for myeloid differentiation. It is now well demonstrated that miRNAs represent a class of genes with a great potential for diagnosis, prognosis and therapy in AML. MicroRNA-based therapy will certainly play a role in the future of treatment of AML.

## Figures and Tables

**Table 1:**
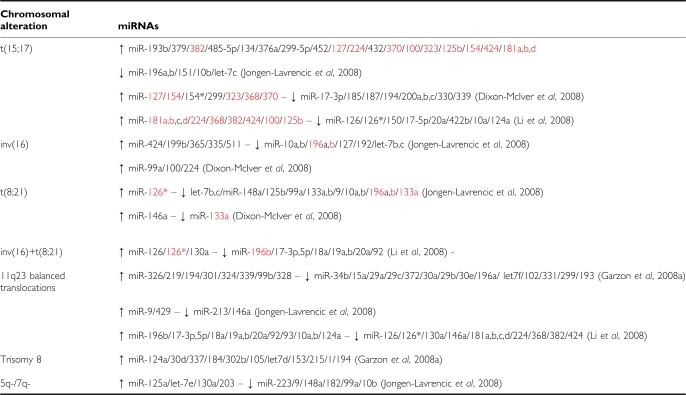
Most significant miRNAs and common miRNAs (in red) deregulated following cytogenetic alterations in AML

**Table 2:**
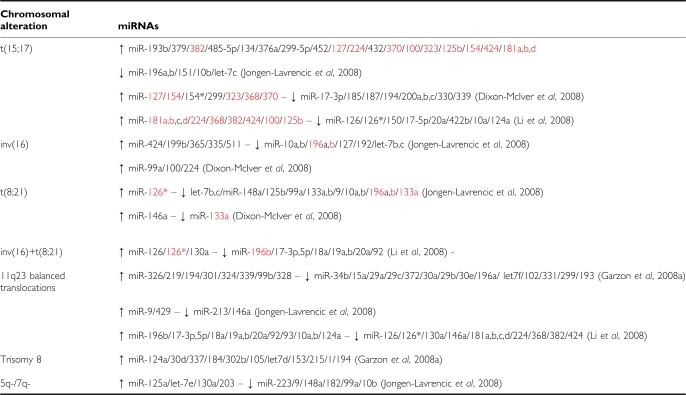
Most significant and common miRNAs (in red) deregulated following molecular alterations in cytogenetically normal AML
